# Long-term exposure to air pollution and incidence risk of various arrhythmias: A prospective cohort study

**DOI:** 10.1016/j.eehl.2024.05.006

**Published:** 2024-06-11

**Authors:** Lu Zhou, Qingli Zhang, Huihuan Luo, Kexin Yu, Xia Meng, Renjie Chen, Haidong Kan

**Affiliations:** a Shanghai Institute of Infectious Disease and Biosecurity, Key Lab of Public Health Safety of the Ministry of Education, NHC Key Lab of Health Technology Assessment, School of Public Health, Fudan University, Shanghai 200032, China; b Children's Hospital of Fudan University, National Center for Children's Health, Shanghai 201102, China

**Keywords:** Air pollution, Atrial fibrillation, Tachycardia, Conduction block, Premature beats, Prospective cohort study

## Abstract

To investigate the association of long-term exposure to air pollution with incident arrhythmia from various causes, this prospective cohort study included 442,386 participants from the UK Biobank cohort. Residential annual average exposures at baseline were evaluated, including fine particles (PM_2.5_), coarse particles (PM_2.5–10_), nitrogen dioxide (NO_2_), and nitrogen oxides (NO_x_). We further constructed a composite air pollution score (APS) to evaluate the concomitant exposure to these four pollutants. The associations of air pollutants with various arrhythmia subtypes were assessed utilizing the Cox proportional hazards model, and the hazard ratios (HRs) for incident arrhythmias were estimated. A total of 41,021 patients with incident arrhythmia were recorded. The HRs of overall arrhythmia associated with a 10 μg/m^3^ increment in PM_2.5_, PM_2.5–10_, NO_2_, and NO_x_ were 1.26, 0.95, 1.03, and 1.02, respectively. The HR was 1.08 in the highest quintile of the APS compared to the lowest one. For cause-specific arrhythmias, the HRs per unit increment in APS were 1.45, 1.67, 1.51, 1.80, 2.63, and 4.66 for atrial fibrillation, atrioventricular block, ventricular fibrillation/tachycardia, intraventricular block, supraventricular tachycardia, and ventricular premature beats, respectively. Females, older individuals, overweight or obese individuals, and those with low education attainment, low income, or cardiometabolic morbidities had higher HRs associated with pollutants. Long-term exposure to air pollution is linked to increased incidence risks of atrial and ventricular arrhythmias. More focus should be shifted to the impact of air pollution on other arrhythmias besides atrial fibrillation.

## Highlights

• Long-term exposures to PM_2.5_, NO_2_, and NO_x_ could elevate arrhythmia incidence risk.
• PM_2.5_ is the most toxic (hazard ratio = 1.26) for arrhythmia, followed by NO_2_ (1.03) and NO_x_ (1.02).
• Arrhythmias other than atrial fibrillation may be more sensitive.

## Graphical abstract

**Figure:**
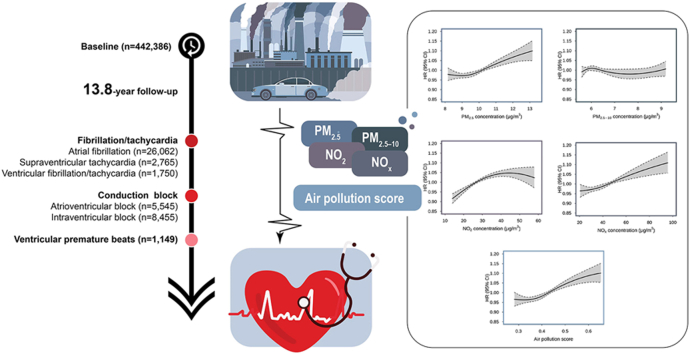

## 1 Introduction

Arrhythmia represents a group of cardiac rhythm disorders, including fibrillation, tachycardia, conduction disorders, and premature beats. Cardiac arrhythmia might induce fatal comorbidities, including heart failure, stroke, myocardial infarction, or sudden death [[1], [2], [3]], consequently affecting quality of life and producing considerable socioeconomic costs [4]. In recent years, the prevalence and burden of arrhythmia, especially atrial fibrillation (AF), have increased among the general adult population worldwide, particularly in more developed regions [2,[5], [6], [7]]. A cardiovascular cohort study in the USA observed a fourfold increment in AF prevalence over half a century of follow-up [7]. The projected AF cases would double in the USA and Europe by the 2050s compared to 2010 [8,9]. Therefore, identifying the modifiable risk factors is crucial for targeted prevention and management of arrhythmia [10,11].

As one of the main contributors to the global disease burden, environmental air pollution is strongly associated with elevated risks of cardiovascular morbidity and mortality [[12], [13], [14]]. Emerging epidemiological studies have suggested the acute effects of air pollution on arrhythmia episodes or exacerbations [[15], [16], [17], [18]], but the chronic effects on the development of arrhythmias remain poorly understood [[18], [19], [20], [21], [22]]. Large-scale cohort-based evidence is essential to establish the causality between air pollutants and arrhythmia. However, the limited cohort studies have generated inconsistent and even controversial findings, which may be due to discrepancies in population characteristics [20,23], sample size [[21], [22], [23], [24]], and outcome ascertainment [22,23]. Furthermore, these studies had focused on AF, and thus, there exists a huge knowledge gap on the long-term associations of air pollutants with other subtypes of arrhythmias [[20], [21], [22], [23], [24], [25], [26]]. Additionally, the associations between particles and arrhythmia were extensively investigated, but the current evidence on gaseous pollutants is still limited and contradictory [17,20,23].

To date, no cohort study has comprehensively assessed the long-term effects of both particulate and gaseous pollutants on incident arrhythmia of various subtypes. Therefore, we conducted this prospective study utilizing the large-scale and well-established UK Biobank cohort, which encompassed sufficient data on systemically investigating the associations of air pollutants with various arrhythmia subtypes. We also investigated the specific subgroups that are more susceptible to the adverse effects.

## 2 Methods

### 2.1 Study design and population

The UK Biobank is a population-based prospective cohort covering over 0.5 million participants aged 40–69 years at enrollment between 2006 and 2010. The data quality has been audited to the highest standards following a consistent protocol accessible online (https://www.ukbiobank.ac.uk/). Briefly, participants were requested to complete touch-screen questionnaires under guidance, take physical measurements, and provide biological samples at assessment centers. Informed written consent was collected from each participant. The UK Biobank Access Committee has granted the authorization for the use of data in this study (application number: 98874).

We initially obtained data of 502,370 participants from the UK Biobank dataset updated to 1st February 2023. For the analysis, we excluded 11,889 participants with arrhythmia or heart failure before enrollment. Then, individuals with missing data on pollutant exposures (n = 40,284) and covariates (n = 7811) were excluded. In total, 442,386 participants were included in the final analyses (Fig. S1).

### 2.2 Outcome ascertainment

We defined health outcomes as incident arrhythmia in this study. Primary or secondary diagnoses for hospital inpatient records in the UK Biobank cohort were determined by linking to the Health Episode Statistics and the Scottish Morbidity Records. We also considered the primary or secondary death cause recorded in death certificates held by the National Health Service Information Centre and the NHS Central Register Scotland. Additionally, we considered the relevant operative procedure information collected in Health Episode Statistics. These electronically linked health-related records were processed and integrated into the UK Biobank data repository after data cleaning, checking, and modification. All diseases were identified according to the 10th revision of the International Classification of Disease (ICD-10) or the Office of Population Censuses and Surveys Classification of Interventions and Procedures (OPCS-4). Detailed definitions for all arrhythmia subtypes, including AF, supraventricular tachycardia, ventricular fibrillation/ventricular tachycardia, atrioventricular block, intraventricular block, and ventricular premature beats, are provided in Table S1.

Besides, participants were followed up from the baseline, from when they first attended the assessment center until the time of the incident event of arrhythmia or censoring. Censoring was defined as the time of loss to follow-up, withdrawal from the study, survival to the end of follow-up (1st February 2023), or death before arrhythmia occurrence, whichever occurred first. If the participants had multiple incident events of different arrhythmia subtypes, then the time of the first event accounted for the overall arrhythmia.

### 2.3 Exposure assessment

The residential annual average concentrations of PM_2.5_, PM_2.5–10_, NO_2_, and NO_x_ were obtained from the UK Biobank dataset between 2005–2007 and 2010. During 2005–2007, air pollution data were derived from Europe-wide air pollution maps [27], from which the UK Biobank extracted pollutant concentrations for each participant by linking the coordinates of the baseline residential address to the corresponding grid cell. Meanwhile, the annual concentrations in 2010 were evaluated through land-use regression (LUR) models. The detailed information and validation of the models have been published elsewhere [28,29]. Generally, LUR is an extensively used technique for modeling spatial variation of air pollution concentrations, incorporating geospatial predictor variables (e.g., traffic intensity, land use, topography) derived from the Geographic Information System (GIS). Specifically, the PM_2.5–10_ concentration was computed as the difference between particles with less than 10 μm in diameter (PM_10_) and PM_2.5_. Notably, the annual concentrations were accessible for several years for NO_2_ (2005–2007 and 2010) and PM_10_ (2007 and 2010), and we used the mean values of multi-year concentrations as the estimates of exposures. Additionally, the annual concentrations of other pollutants in 2010 were directly determined as the corresponding baseline exposures.

Furthermore, we constructed an air pollution score (APS) by summing the concentrations of the four pollutants weighted by the regression coefficients (β) with overall arrhythmia in the single-pollutant models. The method of APS has been described in prior cohort studies [30,31]. The APS was calculated as:

$$APS=\left(\beta_{{PM}_{2.5}}\times {PM}_{2.5}+\beta_{{PM}_{2.5–10}}\times {PM}_{2.5–10}+\beta_{{NO}_{2}}\times {NO}_{2}+\beta_{{NO}_{x}}\times {NO}_{x}\right)\times \left(4/\sum \beta \right)$$

In addition, we subdivided the participants into five groups on the basis of quintiles of the concentration distributions of air pollutants and APS to compare the between-group differences and examine the trends of the risks.

### 2.4 Covariate measurement

In the present study, we identified a series of potential confounders in relation to sociodemographic characteristics, lifestyles, and health status at baseline. They included age, sex, race (white, mixed, Asian, black, Chinese, others), body mass index [BMI, normal (<25 kg/m^2^), overweight (25–30 kg/m^2^), and obese (≥30 kg/m^2^)], Townsend Deprivation index, education level (college degree or above, other levels), average total household income (<£31,000, ≥£31,000), smoking status (never, former or current), alcohol intake frequency (never or occasionally, 1–2 per week, ≥3 per week), healthy diet score (0–5), physical activity level (low, moderate, high), blood pressure, and comorbidities (hypertension, diabetes, disorders of lipoprotein metabolism, heart valve disease, and ischemic heart disease). According to the American Heart Association Guidelines [30], the healthy diet score was calculated by considering the intake frequency of vegetable, fruit, fish, unprocessed red meat, and processed meat. Physical activity level was evaluated through a standard questionnaire [32]. Systolic blood pressure and diastolic blood pressure were measured at baseline with manual or automated readings, mean values of which were used in the analyses. The information on comorbidities was obtained from participants' self-reports or medical records. Additionally, we further included noise pollution [daytime average, evening average, and night-time average noise level (dB)] as environmental confounders.

The proportions of missing data were 20.0%, 15.5%, 2.1%, and 1.5% for physical activity, income, education, and diet score, respectively. For these covariates, we performed random forest regression to impute categorical and continuous variables. We did not consider the issue for other covariates that did not show a missing proportion greater than 1%. Participants were excluded from the analyses if they had missing data on covariates (missing proportion < 1%) in the main models (n = 7811).

In addition to the abovementioned covariates, we performed sensitivity analyses with additional adjustments for several covariates, including coffee and tea intake, sleep quality score, prevalence of respiratory diseases, medication use, and mental health status. The corresponding detailed descriptions of these covariates are presented in the Supplementary Methods. We also described the population characteristics, such as employment and overall health rating, to provide a complete profile of the included participants.

### 2.5 Statistical analyses

We applied Cox proportional hazards models to evaluate the associations of long-term exposures to air pollutants and APS with the incidence of cause-specific arrhythmias. In these models, we controlled for the potential confounders, as follows: age, sex, race, BMI, Townsend Deprivation index, educational level, income, smoking status, alcohol consumption, healthy diet score, physical activity, blood pressure, comorbidities, and environmental noise. The risk estimates were reported as hazard ratios (HRs), and their 95% confidence intervals (CIs) were associated with a 10 μg/m^3^ increment in pollutant concentrations or a unit increment in APS. We applied the Benjamini–Hochberg false discovery rate (FDR) to account for multiple comparisons in assessing the effects of air pollutants on arrhythmia incidence. We utilized a natural cubic spline with 3 degrees of freedom for air pollutants and APS to flexibly illustrate the exposure-response curves.

We performed several stratified analyses to assess potential susceptibility among different subgroups, including age (<60 and ⩾60 years), sex (male and female), BMI (normal, overweight, and obesity), education attainment (college degree or above and other levels), income (<£31,000 and ⩾£31,000), and comorbidities [hypertension, diabetes, disorders of lipoprotein metabolism, valve heart disease, and ischemic heart disease (yes or no)]. The statistical significance of between-group differences was tested by the Z-test. Additionally, we examined the between-group differences between the normal BMI group and the overweight and obesity groups separately.

Several sensitivity analyses were performed separately to test the robustness of the results. First, we further controlled for coffee and tea intake in the model. Second, we adjusted for sleep quality score evaluated by sleep duration, chronotype, insomnia, snoring, and daytime dozing. Third, we controlled for the prevalence of respiratory diseases (emphysema, chronic obstructive pulmonary disease, and asthma) at baseline. Fourth, we adjusted for medication use at baseline, i.e., cholesterol-lowering medication, anti-hypertensive medication, and insulin. Fifth, we controlled for mental health status by the history of general practitioner visits for nerves, anxiety, tension, or depression. Sixth, we constructed the APS after excluding PM_2.5–10_, which showed a non-significant association with incident arrhythmia. Seventh, we excluded participants with incident arrhythmia within the first two years of follow-up. Finally, we limited the follow-up period to the end of 2019 to exclude the potential impact of the COVID-19 pandemic.

All analyses were conducted using R software (version 4.1.0). Multiple imputation and Cox regression were performed using the “mice” and “survival” packages, respectively.

## 3 Results

### 3.1 Descriptive results

In total, 442,386 participants were included in the final analyses, and the baseline characteristics are presented accordingly (Table 1). A total of 41,021 participants were documented to have incident arrhythmia during a median follow-up of 13.8 years. Compared to those without arrhythmia, participants with incident arrhythmia were older, mainly male, and had a higher BMI, lower educational level, or lower income. They were also less likely to have healthy behaviors or lifestyles and more likely to have various comorbidities and poor health status (Table 1 and Table S2). The means [standard deviations (SDs)] of annual average concentrations of PM_2.5_, PM_2.5–10_, NO_2,_ and NO_x_ were 10.00 μg/m^3^ (SD = 1.1), 6.4 μg/m^3^ (SD = 0.9), 29.2 μg/m^3^ (SD = 9.2), and 44.0 μg/m^3^ (SD = 15.6), respectively (Table S3). Additionally, PM_2.5_ and NO_x_ were strongly correlated.

**Table 1:** Summary statistics of participants at baseline in the UK Biobank (n = 442,386).

Variables	Incident arrhythmia cases	
	Yes (n = 41,021)	No (n = 401,365)
Characteristics		
Age (years)	61.3 (6.5)	55.9 (8.1)
Sex, male, n (%)	24,784 (60.4)	174,775 (43.5)
Race, White, n (%)	39,553 (96.4)	378,260 (94.2)
BMI (kg/m^2^)	28.7 (5.2)	27.3 (4.7)
Townsend deprivation index	−1.3 (3.1)	−1.4 (3.0)
Education level, n (%)		
College degree or above	10,733 (26.2)	131,940 (32.9)
Other levels^a^	30,288 (73.8)	269,425 (67.1)
Income, n (%)		
<£31,000	25,820 (62.9)	195,298 (48.7)
≥£31,000	15,201 (37.1)	206,067 (51.3)
**Lifestyle**		
Smoking status, n (%)		
Never	18,964 (46.2)	224,469 (55.9)
Former or current	22,057 (53.8)	176,896 (44.1)
Alcohol consumption frequency, n (%)		
Never or occasionally	18,448 (45.0)	174,585 (43.5)
1–2 per week	13,911 (33.9)	149,525 (37.3)
≥3 per week	8662 (21.1)	77,255 (19.2)
Healthy diet score, n (%)		
0–1	6472 (15.8)	63,274 (15.8)
2–3	23,167 (56.5)	219,324 (54.6)
4–5	11,382 (27.7)	118,767 (29.6)
Physical activity level, n (%)		
Low	8194 (20.0)	76,613 (19.1)
Moderate	16,191 (39.5)	161,809 (40.3)
High	16,636 (40.6)	162,943 (40.6)
**Health status**		
Hypertension, n (%)	17,970 (43.8)	97,159 (24.2)
Diabetes, n (%)	4221 (10.3)	17,794 (4.4)
Disorders of lipoprotein metabolism, n (%)	10,186 (24.8)	53,705 (13.4)
Heart valve disease, n (%)	641 (1.6)	1217 (0.3)
Ischemic heart disease, n (%)	5121 (12.5)	14,741 (3.7)
Systolic blood pressure (mmHg)	145.5 (20.1)	139.2 (19.5)
Diastolic blood pressure (mmHg)	83.0 (11.0)	82.2 (10.6)
**Environmental noise**		
Daytime average level (dB)	55.4 (4.3)	55.4 (4.3)
Evening average level (dB)	51.6 (4.3)	51.7 (4.3)
Night-time average level (dB)	46.6 (4.3)	46.6 (4.3)

BMI, body mass index.

Data are presented as mean (SD) for continuous variables.

a Other levels include advanced levels/advanced subsidiary levels or equivalent, general certificate of education ordinary levels/general certificate of secondary education or equivalent; certificate of secondary education or equivalent; national vocational qualification or higher national diploma or higher national certificate or equivalent; other professional qualifications, e.g., nursing and teaching.

### 3.2 Regression results

**Fig. 1:**
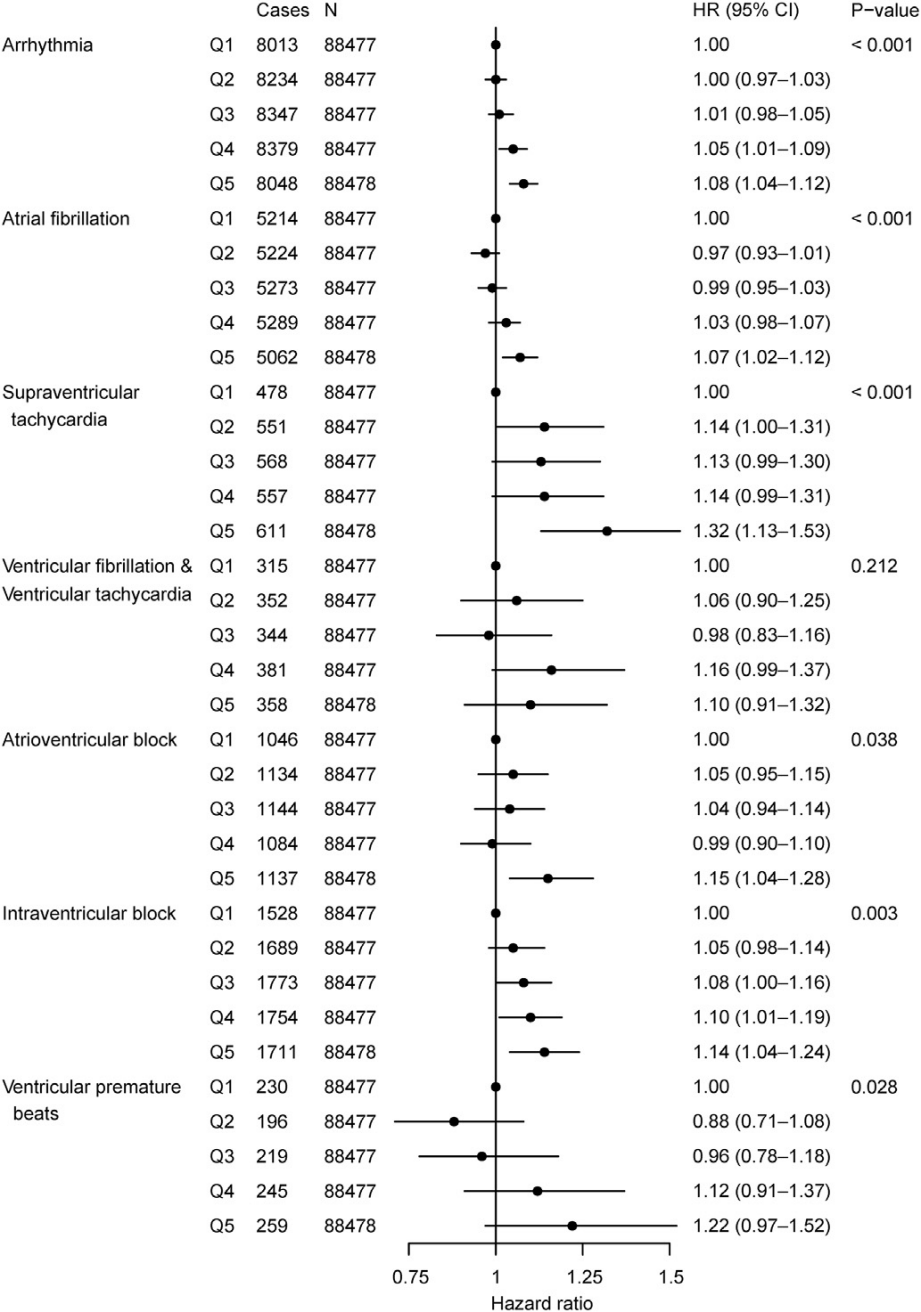
Incidence risks of various arrhythmias associated with air pollution score (in quintiles) in the UK Biobank. Results are presented as hazard ratios and 95% confidence intervals per unit increment for air pollution score.

Long-term exposures to air pollution were significantly associated with increased risks of arrhythmia (Table 2). Generally, PM_2.5_ exhibited the strongest associations with various arrhythmia, followed by NO_2_ and NO_x_. The HRs of overall arrhythmia per 10 μg/m^3^ increment in PM_2.5_, NO_2_, and NO_x_ were 1.26 (95% CI: 1.12–1.41), 1.03 (1.02–1.04), and 1.02 (1.01–1.02), respectively. We observed non-significant associations of PM_2.5–10_ with any type of arrhythmias. When jointly considering all these pollutants, we estimated that one-unit increment in APS corresponded to an HR of 1.52 (1.26–1.83) for overall arrhythmia. As presented in Fig. 1, APS was associated with an increased risk of overall arrhythmia, with HRs increasing from 1.00 (0.97–1.03) at the 2nd quintile to 1.01 (0.98–1.05), 1.05 (1.01–1.09), and 1.08 (1.04–1.12) in the 3rd, 4th and 5th quintile groups (*P* trend < 0.001), compared with the 1st quintile.

**Table 2:** Associations of long-term exposure to air pollutants with the incidence of various arrhythmias in the UK Biobank.

Disease	HR (95% CI)	*P*-value	FDR
**Arrhythmia**			
PM_2.5_	1.26 (1.12–1.41)	<0.001	<0.001
PM_2.5–10_	0.95 (0.84–1.08)	0.505	0.505
NO_2_	1.03 (1.02–1.04)	<0.001	<0.001
NO_X_	1.02 (1.01–1.02)	<0.001	<0.001
Air pollution score	1.52 (1.26–1.83)	<0.001	<0.001
**Atrial fibrillation**			
PM_2.5_	1.21 (1.05–1.40)	0.009	0.014
PM_2.5–10_	0.92 (0.79–1.08)	0.324	0.324
NO_2_	1.03 (1.01–1.04)	0.002	0.005
NO_X_	1.01 (1.00–1.02)	0.014	0.018
Air pollution score	1.45 (1.15–1.84)	0.002	0.005
**Supraventricular tachycardia**			
PM_2.5_	1.46 (0.94–2.27)	0.095	0.158
PM_2.5–10_	1.32 (0.82–2.14)	0.249	0.249
NO_2_	1.11 (1.05–1.17)	<0.001	<0.001
NO_X_	1.03 (0.99–1.06)	0.129	0.161
Air pollution score	2.63 (1.28–5.39)	0.008	0.020
**Ventricular fibrillation & Ventricular tachycardia**			
PM_2.5_	1.15 (0.67–1.98)	0.617	0.788
PM_2.5–10_	1.01 (0.55–1.84)	0.981	0.981
NO_2_	1.05 (0.98–1.12)	0.153	0.765
NO_X_	1.01 (0.97–1.05)	0.630	0.788
Air pollution score	1.51 (0.62–3.67)	0.359	0.788
**Atrioventricular block**			
PM_2.5_	1.38 (1.01–1.89)	0.045	0.098
PM_2.5–10_	1.08 (0.77–1.52)	0.658	0.658
NO_2_	1.03 (1.00–1.07)	0.059	0.098
NO_X_	1.02 (1.00–1.04)	0.118	0.148
Air pollution score	1.67 (1.00–2.79)	0.041	0.098
**Intraventricular block**			
PM_2.5_	1.43 (1.11–1.85)	0.006	0.013
PM_2.5–10_	1.21 (0.91–1.59)	0.184	0.184
NO_2_	1.04 (1.01–1.07)	0.007	0.013
NO_X_	1.02 (1.00–1.04)	0.023	0.029
Air pollution score	1.80 (1.18–2.74)	0.006	0.013
**Ventricular premature beats**			
PM_2.5_	1.97 (1.01–3.84)	0.046	0.077
PM_2.5–10_	0.68 (0.31–1.47)	0.321	0.321
NO_2_	1.15 (1.07–1.24)	<0.001	0.002
NO_X_	1.04 (0.99–1.09)	0.113	0.141
Air pollution score	4.66 (1.60–13.59)	0.005	0.013

Results are presented as HRs and 95% CIs per 10 μg/m^3^ or unit increment for air pollutants and air pollution score, respectively. PM_2.5_, particular matter with an aerodynamic diameter ≤ 2.5 μm; PM_2.5–10_, particular matter with an aerodynamic diameter between 2.5 and 10 μm; NO_2_, nitrogen dioxide; NO_x_, nitrogen oxides; HR, hazard ratio; CI, confidence interval; FDR, false discovery rate.

The risk estimates of air pollution also varied considerably by specific types of arrhythmias (Table 2 and Fig. 1). Generally, the strongest associations were observed for ventricular premature beats and supraventricular tachycardia, followed by an intraventricular block, atrioventricular block, and ventricular fibrillation/ventricular tachycardia, whereas AF exhibited the weakest (but statistically significant) association. For example, the corresponding HRs for a unit increment in APS for these arrhythmia subtypes were 4.66 (1.60–13.59), 2.63 (1.28–5.39), 1.80 (1.18–2.74), 1.67 (1.00–2.79), 1.51 (0.62–3.67), and 1.45 (1.15–1.84). Consistently, for various types of arrhythmias, we found the largest effects of PM_2.5_, followed by NO_2_ and NO_x_. Additionally, none of the air pollutants were significantly associated with ventricular fibrillation/ventricular tachycardia.

**Fig. 2:**
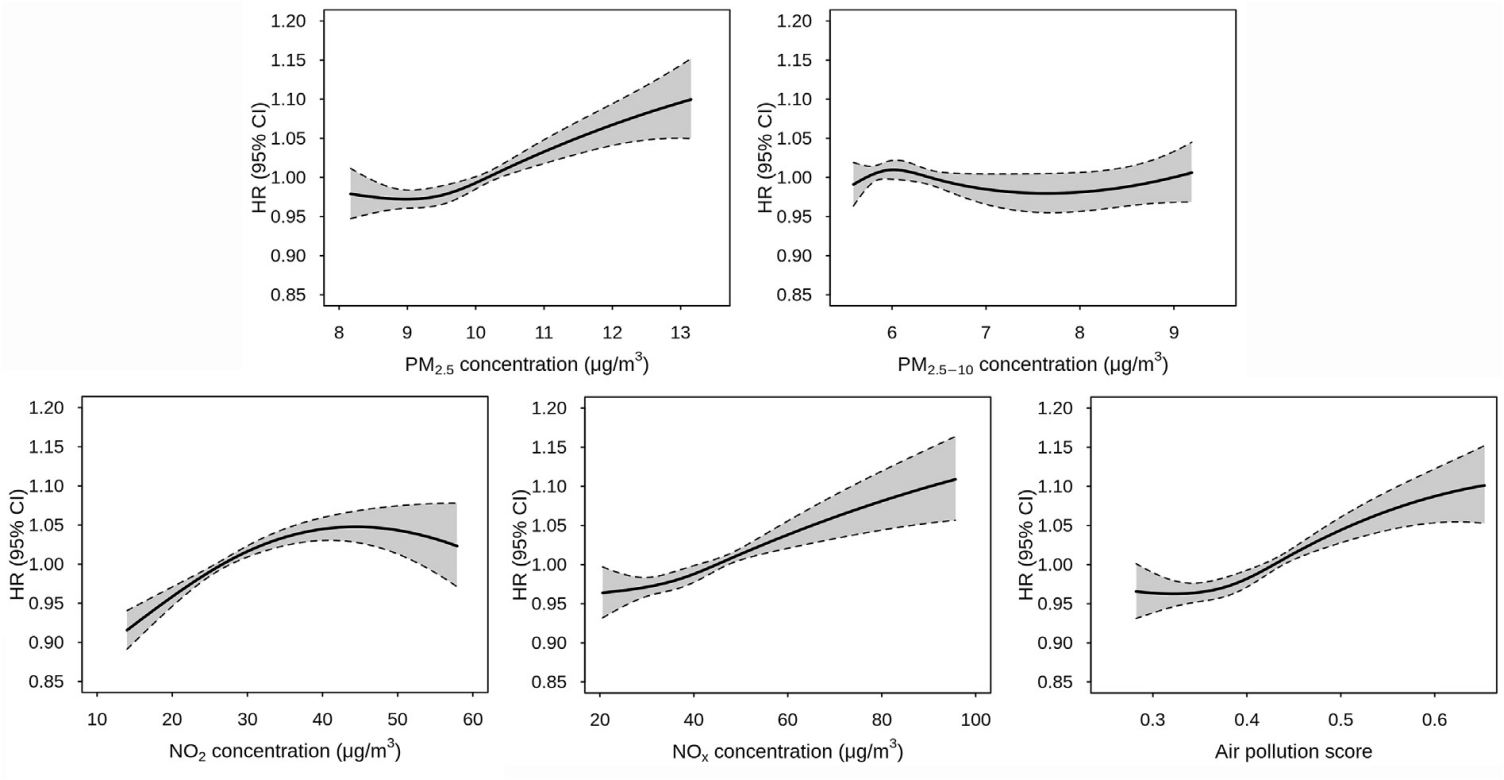
Exposure-response curves for the relationships between air pollution and incidence of arrhythmia. The gray areas denote 95% confidence intervals.

Fig. 2 illustrates the exposure-response curves for relationships between pollutants and overall arrhythmia. Generally, the curve for NO_x_ increased monotonically without apparent threshold; the curve for PM_2.5_ and APS increased at mid-to-high concentrations, and the curve for NO_2_ tended to be flat at higher concentrations. However, there was no clear increase in risk for the curve of PM_2.5–10_. Additionally, the curves for specific subtypes of arrhythmias were slightly different (Figs. S2–S7). For instance, the curves for APS increased at low concentrations for ventricular fibrillation/tachycardia but at medium–high concentrations for atrioventricular block, and the corresponding curves for other subtypes were approximately linear.

**Fig. 3:**
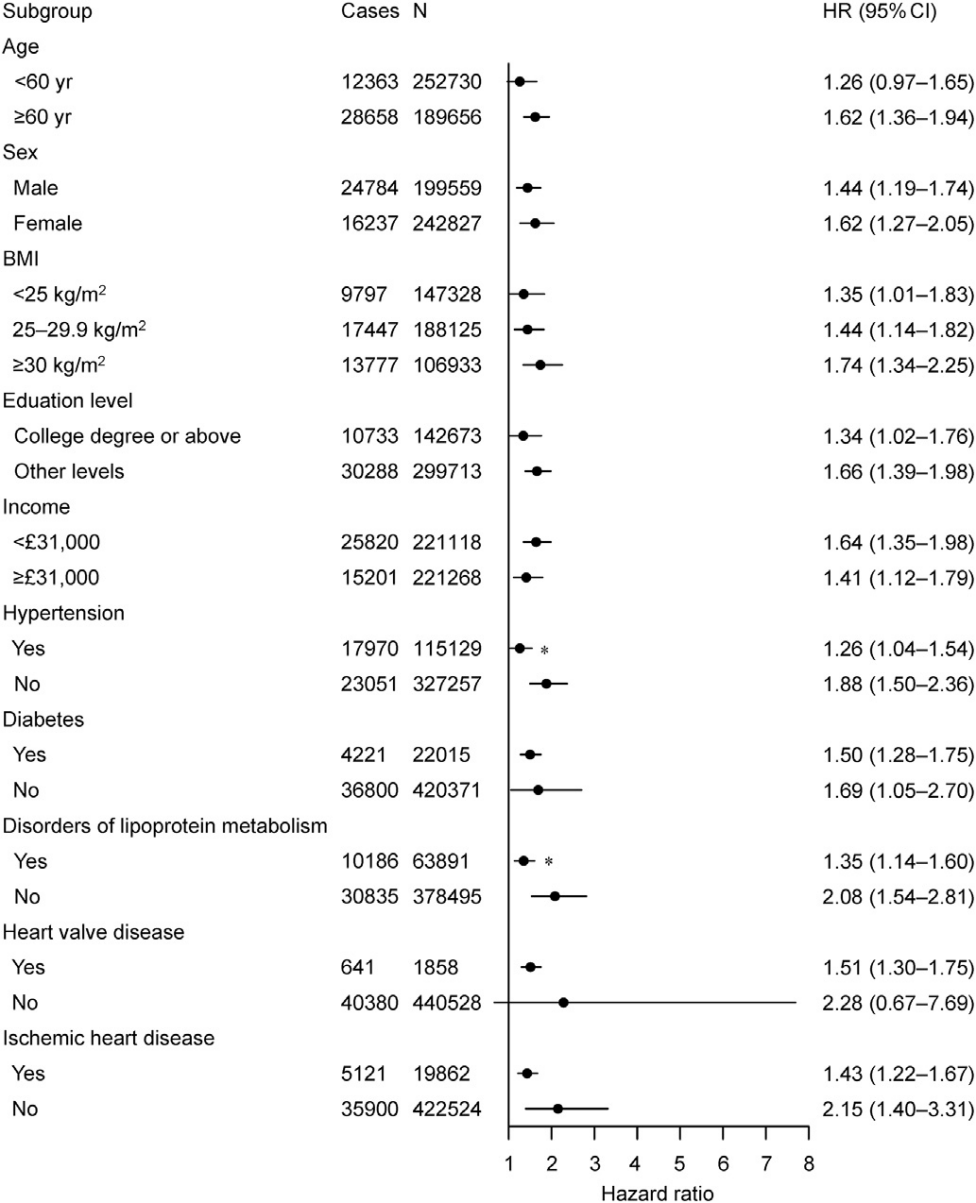
Incidence risk of arrhythmia associated with air pollution in the UK Biobank, stratified by individual characteristics and comorbidities. Results are presented as hazard ratios and 95% confidence intervals for per unit increment for air pollution score. ∗Significant differences between subgroups.

Fig. 3 summarizes the results of stratification analyses. The associations between ambient air pollutants and overall arrhythmia were stronger among those over 60 years old, female, and had a higher BMI, lower educational attainment, or lower income. Additionally, those with comorbidities, including hypertension, diabetes, disorders of lipoprotein metabolism, heart valve disease, or ischemic heart disease, exhibited larger risk estimates (Fig. 3). There were inconsistencies in the statistical significance of the between-group differences across stratified factors and air pollutants (Table S4). For instance, individuals with a college degree were significantly less affected by NO_2_ than their counterparts, but individuals with lower income, hypertension, disorders of lipoprotein metabolism, or ischemic heart disease were significantly more susceptible to NO_2_. Additionally, we did not observe any significant between-group differences for PM_2.5_.

In sensitivity analyses, the association between APS and overall arrhythmia remained robust after further controlling for coffee/tea intake, sleep quality, prevalence of respiratory diseases, medication use, or mental health status at baseline (Table S5). In addition, the risk estimates were barely unchanged after excluding PM_2.5–10_ in APS calculation as well as participants with an incident arrhythmia within the first two-year follow-up, or limiting the follow-up period to the end of 2019.

## 4 Discussion

Among 442,386 participants with a median follow-up of 13.8 years, this cohort study provides the most comprehensive epidemiological evidence on the long-term associations of both PM and gaseous pollutants with cause-specific incident arrhythmias. Overall, our findings demonstrate the detrimental long-term effects of air pollution on various arrhythmia subtypes, among which supraventricular tachycardia and ventricular premature beats were more affected. We highlight the urgency of developing appropriate air pollution abatement measures for vulnerable individuals.

The present cohort study reported an elevated incidence risk of AF associated with PM_2.5_, NO_2_, and NO_x_. Although several observational studies have explored the relationships between air pollutants and AF, existing evidence from prospective cohort studies was limited and inconsistent. For instance, a nationwide Korean cohort study suggested no significant effects of air pollutants on AF occurrence [21]. Another USA cohort study among postmenopausal women reported an elevated risk of AF with long-term exposure to NO_2_ but not to PM_2.5_, PM_10_, or SO_2_ [20]. Additionally, a Danish cohort study suggested the adverse effect of NO_x_ on incident AF [33], whereas another Swedish cohort study did not report such an effect [23]. A recent meta-analysis including several cohort studies estimated an elevated risk of AF with PM_2.5_ (four cohorts, OR = 1.07) and NO_2_ (three cohorts, OR = 1.02) per 10 μg/m^3^ or 10 ppb increment [23]. Our risk estimates were considerably larger than the pooled estimates, which may be explained by higher susceptibility among older adults in the current cohort. Overall, despite the broad inconsistency within the current findings, our cohort study reinforced the existing knowledge that long-term exposure to air pollution can significantly elevate the incidence risk of AF.

A notable strength of this study is the broad exploration of the relationship between long-term air pollution and the incidence of cause-specific arrhythmias. Unlike previous cohort studies focusing on one specific arrhythmia (typically AF), the current study provides ample information on the excess risks of other arrhythmias induced by chronic air pollution exposures. Similar to several cohort studies in South Korea [22], China [34], and the USA [35], we observed a consistent linkage between PM and conduction disorders and also provided novel evidence on the harmful effects of gaseous pollutants. We also provided first-hand cohort-based evidence of the effects of multiple air pollutants on other arrhythmia subtypes, including supraventricular tachycardia and ventricular arrhythmia (tachycardia, fibrillation, and premature beats). There have been conflicting results on the short-term associations of air pollution with these subtypes of arrhythmias, particularly ventricular arrhythmia [19]. For example, a community-based time-series study in China found significant short-term associations of PM_2.5_ with supraventricular arrhythmia, including supraventricular tachycardia, AF, and atrioventricular block, but no significant association with ventricular arrhythmia [36]. In contrast, some other investigations reported an elevated risk of ventricular arrhythmia linked to short-term exposures to air pollution [17,[37], [38], [39]]. Overall, further cohort studies are required to verify the positive associations of long-term air pollution exposures with various incident arrhythmias. Furthermore, we observed considerably smaller chronic effects of pollutants on incident AF compared to other subtypes of arrhythmias, which is in line with previous investigations on the short-term effects [15,36]. Overall, our findings suggest that there exist arrhythmia subtypes other than AF that could be more vulnerable to air pollution exposures; thus, a comprehensive risk assessment, rather than focusing solely on AF, is needed to improve the primary prevention and control of arrhythmias.

Our results on air pollution chronic exposure and incident arrhythmia are biologically plausible. A wealth of mechanistic evidence has indicated oxidative stress, systemic inflammation, endothelial dysfunction, and autonomic dysregulation following air pollution exposure [17,18]. These biological processes play critical roles in the formation of arrhythmia [13,18]. Our findings suggest that the magnitude of risk estimates was strongest for PM_2.5_, followed by NO_2_ and NO_x_. This is plausible because of the well-documented cardiovascular toxicity of PM_2.5_ and the established causality between PM_2.5_ and cardiac autonomic dysfunction, compared to other pollutants [18,23]. The current study found a non-significant association between PM_2.5–10_ and incident arrhythmia, which is largely consistent with prior findings on PM_2.5–10_ and cardiovascular outcomes [18,30]. These findings are reasonable because PM_2.5_ is primarily derived from traffic- or industry-related fossil fuel combustion with more toxic constituents and deeper respiratory deposition compared with PM_2.5–10_ [13]. Furthermore, our findings reveal significant effects of air pollution on incident arrhythmia across various subtypes. Ventricular premature beats, tachycardia, and conduction disorders might be primarily induced by autonomic dysfunction, marked by a decrease in parasympathetic tone and compensatory activation of sympathetic tone [34,39]. Meanwhile, disturbed electrophysiological activities and cardiac structural remodeling (e.g., fibrosis) are suggested as one of the underlying pathways whereby air pollution induces AF [16]. Currently, the exact mechanisms of the differential effects on cause-specific arrhythmias are not yet fully understood. Further research is warranted to determine the specific mechanisms underlying the associations between air pollution and various subtypes of arrhythmias [18,40].

Consistent with previous studies, we found a higher incidence risk among older individuals [22,23,25,34], females [23,26], and those with higher BMI [34], lower educational levels, lower income [26], or comorbidities [23,26,34]. We found no significant association between air pollution (except for PM_2.5_) and incident arrhythmia among individuals aged less than 60 years, consistent with previous cohort studies conducted in South Korea [21] and Sweden [24]. Although the between-group differences were nonsignificant across air pollutants for age subgroups, older individuals showed consistently higher risks than younger individuals, which might be explained by a higher prevalence of predisposed cardiometabolic risks and pre-existing decline of cardiac autonomic function with age. Particularly, individuals with cardiometabolic comorbidities have heightened oxidative stress and impaired oxidant defenses [41,42], which also contribute to potential susceptibility. Besides, the higher risks of females and individuals with lower educational levels or lower income could be due to structural disadvantages in terms of social determinants [43]. Therefore, more targeted intervention strategies are needed to address the health inequalities related to air pollution.

Several limitations should be acknowledged. First, although we assigned residential air pollutant concentrations using exposure models of high spatial resolution, exposure misclassification is still inevitable because we did not consider the exposures in indoor environments and throughout the follow-up periods. Second, the confounding variables included in the present analyses were only measured at baseline, which did not account for their potential impacts of time-varying changes on incident arrhythmia. Third, we used available datasets in the UK Biobank to diagnose the incidence of arrhythmias, but we might still underestimate the number of incident cases due to the limited use of dynamic monitoring and the prevalence of asymptomatic arrhythmias [44], and the potential diagnostic misclassification derived from administrative health data is inevitable. Fourth, we did not assess other air pollutants (e.g., ozone, sulfur dioxide, and nitric oxide) in the current study because the exposure data were not publicly available in the UK Biobank dataset. Finally, the participants included in the UK Biobank are predominantly white individuals from the UK. Thus, our findings might not be generalizable to other populations with different genetic characteristics and socioeconomic backgrounds. Future studies should integrate dynamic air pollution data covering multiple pollutants over time to accurately assess their long-term effects on arrhythmias. Furthermore, the integration of evidence from diverse cohorts across various populations and countries is crucial for a comprehensive understanding. These endeavors are critical to informing evidence-based public health interventions and policies geared towards ameliorating the air pollution-related arrhythmias burden worldwide.

In summary, this study provides convincing evidence that long-term exposure to air pollution can increase the incidence of various arrhythmias. We identified that PM_2.5_ is more hazardous than other air pollutants, and specific arrhythmias other than AF (particularly, ventricular premature beats and supraventricular tachycardia) could be more sensitive to air pollution exposures. Furthermore, the risks were slightly higher for the elders, females, overweight or obese individuals, and those with lower educational attainment, lower income, or pre-existing cardiometabolic comorbidities. Overall, this study underlines the significance of air pollution exposures in the development of various arrhythmias and is helpful for designing tailored intervention strategies for incident arrhythmias.

## CRediT authorship contribution statement

**Lu Zhou:** Conceptualization, Data curation, Formal analysis, Methodology, Writing – original draft. **Qingli Zhang:** Data curation, Methodology, Validation. **Huihuan Luo:** Data curation, Methodology, Validation. **Kexin Yu:** Data curation. **Xia Meng:** Data curation, Writing – review & editing. **Renjie Chen:** Conceptualization, Supervision, Validation, Writing – review & editing, Project administration. **Haidong Kan:** Conceptualization, Funding acquisition, Supervision, Validation, Writing – review & editing, Project administration.

## Declaration of competing interests

The authors have declared no conflicts of interest.
