# Supplementary material for: Long-term exposure to air pollution and incidence risk of various arrhythmias: A prospective cohort study
Source: Eco Environ Health. 2024 Jun 11;3(4):445–51. doi: 10.1016/j.eehl.2024.05.006 (PMC11570411; doi:10.1016/j.eehl.2024.05.006)
Supplement: Multimedia component 1 [file mmc1.docx]

**Supporting information**

**Long-term exposure to air pollution and incidence risk of various arrhythmias: a prospective cohort study**

**Supplementary Methods**

**Table S1.** Arrhythmia definitions.

**Table S2.** Additional statistics of participants in the UK Biobank.

**Table S3.** Summary statistics of air pollution levels and Pearson’s correlation coefficients in the UK Biobank.

**Table S4.** Summary for the association between air pollution and incident overall arrhythmia in the UK Biobank, stratified by individual-level characteristics.

**Table S5.** The results of sensitivity analyses on the association between air pollution and incident overall arrhythmia in the UK Biobank.

**Fig. S1.** Flowchart of study inclusion and exclusion criteria.

**Fig. S2.** Exposure-response curves for the relationships between air pollution and incidence of atrial fibrillation.

**Fig. S3.** Exposure-response curves between air pollution and incidence of supraventricular tachycardia.

**Fig. S4.** Exposure-response curves between air pollution and incidence of ventricular fibrillation and ventricular tachycardia.

**Fig. S5.** Exposure-response curves between air pollution and incidence of atrioventricular block.

**Fig. S6.** Exposure-response curves between air pollution and incidence of intraventricular block.

**Fig. S7.** Exposure-response curves between air pollution and incidence of ventricular premature beats.

**Supplementary Methods**

***Covariate Measurement***

We evaluated coffee and tea intake through UKB’s touchscreen questionnaire on diet thorough data fields including coffee intake (Field ID: 1498; "How many cups of coffee do you drink each DAY? (Include decaffeinated coffee)") and tea intake (Field ID: 1498; "How many cups of tea do you drink each DAY? (Include black and green tea)"). We classified the intake into <= 3 cups/d, 3–6 cups/d, and > 6 cups/d.

We constructed a sleep quality score that integrates five aspects (sleep duration, chronotype, insomnia, snoring and daytime dozing) to assess sleep quality in the present study. Sleeping 7–8 hour per day, having an early chronotype, nerve or rarely having insomnia symptoms, not reporting snoring, and not reporting daytime dozing were considered healthy sleep behaviors with a score of 1. The scores for all five factors were added to calculate a sleep quality score and higher scores represent higher sleep quality.

Participants who had a history of at least one of the following respiratory diseases: emphysema, chronic obstructive pulmonary disease, and asthma were identified as the individuals with respiratory diseases. Participants who had taken at least one of the following medications: cholesterol lowering medication, blood pressure medication, and insulin were classified as the medication use group.

For mental health assessment, we applied the history of general practitioner visits for nerves, anxiety, tension, or depression (Field ID: 2090; "Have you ever seen a general practitioner (GP) for nerves, anxiety, tension or depression?") at baseline to identified the participants who experienced mental health issues.

**Table S1.** Arrhythmia definitions.

|  | **Source** | **Codes** |
| --- | --- | --- |
|  |  |  |
| **Arrhythmia** | Diagnosis or Death (ICD-10 codes main or secondary) | I44, I45, I47, I48, I49 |
|  | Operative Procedures (OPCS4 code main or secondary) | K57.6, K59, K60, K61, K62, K64.1,  K72, K73, K74 |
| Atrial fibrillation/flutter | Diagnosis or Death (ICD-10 codes main or secondary) | I48 |
|  | Operative Procedures (OPCS4 code main or secondary) | K62 |
| Supraventricular tachycardia | Diagnosis or Death (ICD-10 codes main or secondary) | I47.1 |
| Ventricular fibrillation &  Ventricular tachycardia | Diagnosis or Death (ICD-10 codes main or secondary) | I47.2, I49.0 |
|  | Operative Procedures (OPCS4 code main or secondary) | K57.6, K64.1 |
| Atrioventricular block | Diagnosis or Death (ICD-10 codes main or secondary) | I44.0, I44.1, I44.2, I44.3, I45.8 |
| Intraventricular block | Diagnosis or Death (ICD-10 codes main or secondary) | I44.4, I44.5, I44.6, I44.7, I45.0, I45.1, I45.2, I45.3, I45.4 |
| Ventricular premature beats | Diagnosis or Death (ICD-10 codes main or secondary) | I49.3 |

**Table S2. Additional statistics of participants in the UK Biobank (n=442,386).**

| **Variables** | **Incident arrhythmia cases** | |
| --- | --- | --- |
|  | **Yes (n=41,021)** | **No (n=401,365)** |
| **Characteristics** |  |  |
| Employment* |  |  |
| Job involves mainly walking or standing, n (%) |  |  |
| Never or rarely | 4,860 (11.8) | 85,198 (21.2) |
| Sometimes | 5,111 (12.5) | 73,154 (18.2) |
| Usually or always | 5,967 (14.5) | 81,468 (20.3) |
| Missing | 25,083 (61.1) | 161,545 (40.2) |
| Job involves heavy manual/physical work, n (%) |  |  |
| Never or rarely | 9,610 (23.4) | 156,084 (38.9) |
| Sometimes | 3,799 (9.3) | 51,433 (12.8) |
| Usually or always | 2,536 (6.2) | 32,339 (8.1) |
| Missing | 25,076 (61.1) | 161,509 (40.2) |
| Job involves shift work, n (%) |  |  |
| Never or rarely | 12,989 (31.7) | 198,690 (49.5) |
| Sometimes | 1,270 (3.1) | 17,431 (4.3) |
| Usually or always | 1,665 (4.1) | 23,427 (5.8) |
| Missing | 25,097 (61.2) | 161,807 (40.3) |
| **Lifestyle** |  |  |
| Coffee intake, n (%) |  |  |
| ≤3 cups/d | 32,707 (79.7) | 322,900 (80.4) |
| 3–6 cups/d | 7,058 (17.2) | 66,990 (16.7) |
| >6 cups/d | 1,256 (3.1) | 11,475 (2.9) |
| Tea intake, n (%) |  |  |
| ≤3 cups/d | 21,762 (53.1) | 222,230 (55.4) |
| 3–6 cups/d | 14,689 (35.8) | 139,304 (34.7) |
| >6 cups/d | 4,570 (11.1) | 39,831 (9.9) |
| Sleep quality score, n (%) |  |  |
| 0–1 | 8,194 (20.0) | 76,613 (19.1) |
| 2–3 | 16,191 (39.5) | 161,809 (40.3) |
| 4–5 | 16,636 (40.6) | 162,943 (40.6) |
| **Health status** |  |  |
| Overall health rating* |  |  |
| Poor, n (%) | 3,133 (7.6) | 15,302 (3.8) |
| Fair, n (%) | 11,568 (28.2) | 80,110 (20.0) |
| Good, n (%) | 21,732 (53.0) | 236,530 (58.9) |
| Excellent, n (%) | 4,373 (10,7) | 67,714 (16.9) |
| Missing, n (%) | 215 (0.5) | 1,709 (0.4) |
| Respiratory diseases, n (%) | 6,326 (15.4) | 50,085 (12.5) |
| Asthma, n (%) | 5,493 (13.4) | 465,85 (11.6) |
| Emphysema or chronic obstructive  pulmonary disease, n (%) | 1,613 (3.9) | 6,224 (1.6) |
| Medication use, n (%) | 19,436 (47.4) | 97,816 (24.4) |
| Mental health issues, n (%) | 10,186 (24.8) | 53,705 (13.4) |

Data are presented as mean (SD) for continuous variables.

*Covariates that were not included in the analyses, and we did not apply multiple imputation for their missing values.

**Table S3.** Summary statistics of air pollution levels and Pearson’s correlation coefficients in the UK Biobank.

| **Pollutant** | **Mean** | **SD** | **Minimum** | **Maximum** | **IQR** | **Pearson’s correlation coefficient** | | | |  |
| --- | --- | --- | --- | --- | --- | --- | --- | --- | --- | --- |
|  |  |  |  |  |  | **PM_2.5_** | **PM_2.5–10_** | **NO_2_** | **NO_X_** | **APS** |
| PM_2.5_ | 10.0 | 1.1 | 8.2 | 21.3 | 1.3 | 1.00 | - | - | - | - |
| PM_2.5–10_ | 6.4 | 0.9 | 5.6 | 12.8 | 0.8 | 0.22* | 1.00 | - | - | - |
| NO_2_ | 29.2 | 9.2 | 8.9 | 125.1 | 11.0 | 0.73* | 0.18* | 1.00 | - | - |
| NO_X_ | 44.0 | 15.6 | 19.7 | 265.9 | 16.5 | 0.85* | 0.24* | 0.75* | 1.00 | - |
| APS | 0.35 | 0.07 | 0.20 | 1.12 | 0.08 | 0.93* | 0.17* | 0.90* | 0.93* | 1.00 |

**Abbreviations:** SD, standard deviation; IQR, interquartile range; PM_2.5_, particular matter with an aerodynamic diameter ≤ 2.5 μm; PM_2.5–10_, particular matter with an aerodynamic diameter between 2.5 and 10 μm; NO_2_, nitrogen dioxide; NO_x_, nitrogen oxides; APS, air pollution score.

*P-value <0.001

**Table S4.** Summary for the association between air pollution and incident overall arrhythmia in the UK Biobank, stratified by individual-level characteristics.

|  | **HR (95% CI)** | **P-value** | **^*^Between-group p-value** |
| --- | --- | --- | --- |
|  |  |  |  |
| **PM_2.5_** |  |  |  |
| Age <60 y | 1.22 (1.01–1.47) | 0.039 | 0.477 |
| Age ≥60 y | 1.32 (1.17–1.50) | <0.001 |  |
| Male | 1.23 (1.08–1.41) | 0.002 | 0.333 |
| Female | 1.37 (1.16–1.63) | <0.001 |  |
| BMI <25 kg/m^2^ | 1.24 (1.00–1.54) | 0.052 | Ref |
| BMI =25–29.9 kg/m^2^ | 1.28 (1.09–1.50) | 0.003 | 0.823 |
| BMI ≥30 kg/m^2^ | 1.35 (1.13–1.62) | 0.001 | 0.544 |
| College degree or above | 1.28 (1.05–1.57) | 0.017 | 0.810 |
| Other levels | 1.32 (1.17–1.49) | <0.001 |  |
| Income <£31,000 | 1.29 (1.13–1.48) | <0.001 | 0.808 |
| Income ≥£31,000 | 1.33 (1.12–1.58) | 0.001 |  |
| Without hypertension | 1.19 (1.03–1.37) | 0.018 | 0.087 |
| With hypertension | 1.43 (1.22–1.67) | <0.001 |  |
| Without diabetes | 1.28 (1.15–1.43) | <0.001 | 0.595 |
| With diabetes | 1.41 (1.01–1.96) | 0.041 |  |
| Without DLM | 1.23 (1.08–1.38) | 0.001 | 0.120 |
| With DLM | 1.49 (1.20–1.84) | 0.001 |  |
| Without VHD | 1.29 (1.16–1.43) | <0.001 | 0.495 |
| With VHD | 1.73 (0.75–3.96) | 0.199 |  |
| Without IHD | 1.27 (1.13–1.42) | <0.001 | 0.549 |
| With IHD | 1.40 (1.04–1.88) | 0.026 |  |
| **NO_2_** |  |  |  |
| Age <60 y | 1.02 (0.99–1.04) | 0.163 | 0.075 |
| Age ≥60 y | 1.04 (1.03–1.06) | <0.001 |  |
| Male | 1.03 (1.02–1.05) | <0.001 | 0.840 |
| Female | 1.04 (1.02–1.06) | <0.001 |  |
| BMI <25 kg/m^2^ | 1.02 (1.00–1.05) | 0.066 | Ref |
| BMI =25–29.9 kg/m^2^ | 1.03 (1.01–1.05) | 0.008 | 0.838 |
| BMI ≥30 kg/m^2^ | 1.05 (1.03–1.08) | <0.001 | 0.079 |
| College degree or above | 1.01 (0.99–1.04) | 0.235 | 0.008 |
| Other levels | 1.05 (1.03–1.07) | <0.001 |  |
| Income <£31,000 | 1.05 (1.03–1.07) | <0.001 | 0.023 |
| Income ≥£31,000 | 1.02 (1.00–1.04) | 0.049 |  |
| Without hypertension | 1.02 (1–1.04) | 0.024 | 0.008 |
| With hypertension | 1.05 (1.03–1.07) | <0.001 |  |
| Without diabetes | 1.03 (1.02–1.05) | <0.001 | 0.353 |
| With diabetes | 1.05 (1.01–1.09) | 0.010 |  |
| Without DLM | 1.02 (1.01–1.04) | 0.002 | 0.004 |
| With DLM | 1.07 (1.04–1.09) | <0.001 |  |
| Without VHD | 1.03 (1.02–1.05) | <0.001 | 0.513 |
| With VHD | 1.07 (0.97–1.18) | 0.190 |  |
| Without IHD | 1.03 (1.01–1.04) | <0.001 | 0.002 |
| With IHD | 1.09 (1.05–1.13) | <0.001 |  |
| **NO_X_** |  |  |  |
| Age <60 y | 1.01 (0.99–1.02) | 0.260 | 0.079 |
| Age ≥60 y | 1.02 (1.01–1.03) | <0.001 |  |
| Male | 1.02 (1.01–1.03) | 0.004 | 0.332 |
| Female | 1.02 (1.01–1.04) | <0.001 |  |
| BMI <25 kg/m^2^ | 1.01 (1.00–1.03) | 0.115 | Ref |
| BMI =25–29.9 kg/m^2^ | 1.02 (1.01–1.03) | 0.004 | 0.614 |
| BMI ≥30 kg/m^2^ | 1.02 (1.01–1.04) | <0.001 | 0.359 |
| College degree or above | 1.02 (1.00–1.03) | 0.025 | 0.708 |
| Other levels | 1.02 (1.01–1.03) | <0.001 |  |
| Income <£31,000 | 1.02 (1.01–1.03) | 0.007 | 0.841 |
| Income ≥£31,000 | 1.02 (1.01–1.03) | 0.006 |  |
| Without hypertension | 1.01 (1.00–1.02) | 0.126 | 0.005 |
| With hypertension | 1.03 (1.02–1.04) | <0.001 |  |
| Without diabetes | 1.02 (1.01–1.03) | <0.001 | 0.668 |
| With diabetes | 1.01 (0.99–1.04) | 0.284 |  |
| Without DLM | 1.01 (1.00–1.02) | 0.004 | 0.044 |
| With DLM | 1.03 (1.02–1.05) | <0.001 |  |
| Without VHD | 1.02 (1.01–1.03) | <0.001 | 0.680 |
| With VHD | 1.03 (0.97–1.10) | 0.331 |  |
| Without IHD | 1.02 (1.01–1.03) | <0.001 | 0.325 |
| With IHD | 1.03 (1.01–1.05) | 0.012 |  |
| **Air pollution score** |  |  |  |
| Age <60 y | 1.26 (0.97–1.65) | 0.087 | 0.123 |
| Age ≥60 y | 1.62 (1.36–1.94) | <0.001 |  |
| Male | 1.44 (1.19–1.74) | <0.001 | 0.450 |
| Female | 1.62 (1.27–2.05) | <0.001 |  |
| BMI <25 kg/m^2^ | 1.35 (1.01–1.83) | 0.046 | Ref |
| BMI =25–29.9 kg/m^2^ | 1.44 (1.14–1.82) | 0.002 | 0.747 |
| BMI ≥30 kg/m^2^ | 1.74 (1.34–2.25) | <0.001 | 0.214 |
| College degree or above | 1.34 (1.02–1.76) | 0.035 | 0.201 |
| Other levels | 1.66 (1.39–1.98) | <0.001 |  |
| Income <£31,000 | 1.64 (1.35–1.98) | <0.001 | 0.353 |
| Income ≥£31,000 | 1.41 (1.12–1.79) | 0.004 |  |
| Without hypertension | 1.26 (1.04–1.54) | 0.020 | 0.009 |
| With hypertension | 1.88 (1.50–2.36) | <0.001 |  |
| Without diabetes | 1.50 (1.28–1.75) | <0.001 | 0.647 |
| With diabetes | 1.69 (1.05–2.70) | 0.030 |  |
| Without DLM | 1.35 (1.14–1.60) | <0.001 | 0.014 |
| With DLM | 2.08 (1.54–2.81) | <0.001 |  |
| Without VHD | 1.51 (1.30–1.75) | <0.001 | 0.509 |
| With VHD | 2.28 (0.67–7.69) | 0.186 |  |
| Without IHD | 1.43 (1.22–1.67) | <0.001 | 0.079 |
| With IHD | 2.15 (1.40–3.31) | <0.001 |  |

**Abbreviations:** PM_2.5_, particular matter with an aerodynamic diameter ≤ 2.5 μm; PM_2.5–10_, particular matter with an aerodynamic diameter between 2.5 and 10 μm; NO_2_, nitrogen dioxide; NO_x_, nitrogen oxides; BMI, body mass index; DLM, disorders of lipoprotein metabolism; VHD, valve heart disease; IHD, ischemic heart disease; HR, hazard ratio; CI, confidence interval.

Results are presented as hazard ratios and 95% confidence intervals for per 10 μg/m^3^ or unit increment for air pollutants and air pollution score, respectively.

^*^The between-group p-values were derived by examining the between-group differences in incident arrhythmia associated with air pollutants and air pollution score in stratification analyses by age, sex, BMI, education level, income level, and prevalent comorbidities.

**Table S5.** The results of sensitivity analyses on the association between air pollution and incident overall arrhythmia in the UK Biobank.

|  | **Air pollution score (quintiles)** | | | | |  | **P-trend** | **Hazard Ratio for**  **per unit increment** | **P-value** |
| --- | --- | --- | --- | --- | --- | --- | --- | --- | --- |
|  | **Q1** | **Q2** | **Q3** | **Q4** | **Q5** |  |  |  |  |
| **After including several covariates** | | | | | | | | | |
| Model 1^a^ | 1.00 | 1.01 (0.97–1.04) | 1.01 (0.98–1.05) | 1.05 (1.01–1.09) | 1.08 (1.04–1.12) |  | <0.001 | 1.52 (1.26–1.83) | <0.001 |
| Model 2^b^ | 1.00 | 1.00 (0.97–1.03) | 1.01 (0.98–1.05) | 1.05 (1.01–1.08) | 1.08 (1.03–1.12) |  | <0.001 | 1.52 (1.25–1.85) | <0.001 |
| Model 3^c^ | 1.00 | 1.00 (0.97–1.04) | 1.01 (0.98–1.05) | 1.05 (1.01–1.09) | 1.08 (1.04–1.12) |  | <0.001 | 1.52 (1.26–1.84) | <0.001 |
| Model 4^d^ | 1.00 | 1.01 (0.97–1.06) | 0.99 (0.95–1.03) | 1.05 (1.01–1.10) | 1.07 (1.02–1.12) |  | 0.003 | 1.60 (1.18–2.19) | 0.003 |
| Model 5^e^ | 1.00 | 1.01 (0.97–1.04) | 1.01 (0.98–1.05) | 1.05 (1.01–1.08) | 1.07 (1.03–1.12) |  | <0.001 | 1.50 (1.24–1.82) | <0.001 |
| **After excluding PM_2.5–10_ in air pollution score calculation** | | | | | | | | | |
| Model 7^f^ | 1.00 | 1.00 (0.97–1.04) | 1.01 (0.97–1.04) | 1.04 (1.01–1.08) | 1.07 (1.03–1.12) |  | <0.001 | 1.51 (1.25–1.81) | <0.001 |
| **After excluding participants with incident arrhythmia within the first 2 year of follow-up** | | | | | | | | | |
| Model 8^f^ | 1.00 | 1.00 (0.96–1.04) | 1.00 (0.97–1.04) | 1.05 (1.01–1.09) | 1.07 (1.03–1.12) |  | <0.001 | 1.52 (1.24–1.87) | <0.001 |
| **After limiting the follow-up period to the end of 2019 (before the COVID-19 outbreak)** | | | | | | | | | |
| Model 8^f^ | 1.00 | 1.02 (0.99–1.06) | 1.04 (1.00–1.08) | 1.07 (1.03–1.11) | 1.11 (1.06–1.16) |  | <0.001 | 1.55 (1.29–1.87) | <0.001 |

^a^ Adjusted for age, sex, race, body mass index category, Townsend Deprivation index category, education level, average total household income, smoking status, alcohol consumption frequency, healthy diet score, physical activity, noise pollution, comorbidities (hypertension, diabetes, disorders of lipoprotein metabolism, heart valve disease, ischemic heart disease), systolic blood pressure, diastolic blood pressure, coffee intake, and tea intake.

^b^ Adjusted for age, sex, race, body mass index category, Townsend Deprivation index category, education level, average total household income, smoking status, alcohol consumption frequency, healthy diet score, physical activity, noise pollution, comorbidities (hypertension, diabetes, disorders of lipoprotein metabolism, heart valve disease, ischemic heart disease), systolic blood pressure, diastolic blood pressure, and sleep quality.

^c^ Adjusted for age, sex, race, body mass index category, Townsend Deprivation index category, education level, average total household income, smoking status, alcohol consumption frequency, healthy diet score, physical activity, noise pollution, comorbidities (hypertension, diabetes, disorders of lipoprotein metabolism, heart valve disease, ischemic heart disease), systolic blood pressure, diastolic blood pressure, and respiratory diseases (emphysema, chronic obstructive pulmonary disease, and asthma).

^d^ Adjusted for age, sex, race, body mass index category, Townsend Deprivation index category, education level, average total household income, smoking status, alcohol consumption frequency, healthy diet score, physical activity, noise pollution, comorbidities (hypertension, diabetes, disorders of lipoprotein metabolism, heart valve disease, ischemic heart disease), systolic blood pressure, diastolic blood pressure, and medication use (cholesterol lowering medication, anti-hypertensive medication, and insulin).

^e^ Adjusted for age, sex, race, body mass index category, Townsend Deprivation index category, education level, average total household income, smoking status, alcohol consumption frequency, healthy diet score, physical activity, noise pollution, comorbidities (hypertension, diabetes, disorders of lipoprotein metabolism, heart valve disease, ischemic heart disease), systolic blood pressure, diastolic blood pressure, and mental health status.

^f^ Adjusted for age, sex, race, body mass index category, Townsend Deprivation index category, education level, average total household income, smoking status, alcohol consumption frequency, healthy diet score, physical activity, noise pollution, comorbidities (hypertension, diabetes, disorders of lipoprotein metabolism, heart valve disease, ischemic heart disease), systolic blood pressure, and diastolic blood pressure.

Results are presented as hazard ratios and 95% confidence intervals.

**
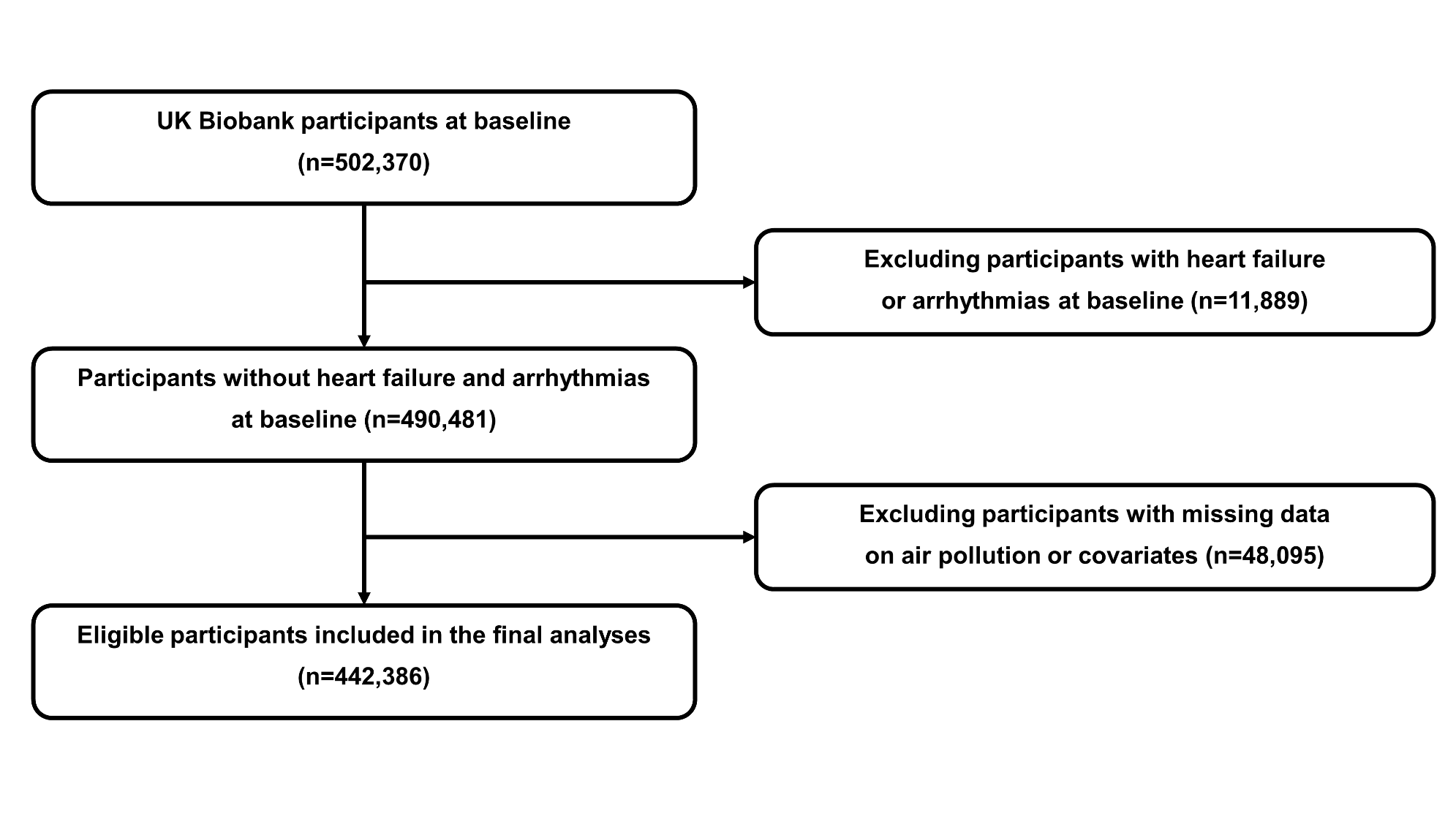
Fig. S1.** Flowchart of participant inclusion and exclusion criteria.

**
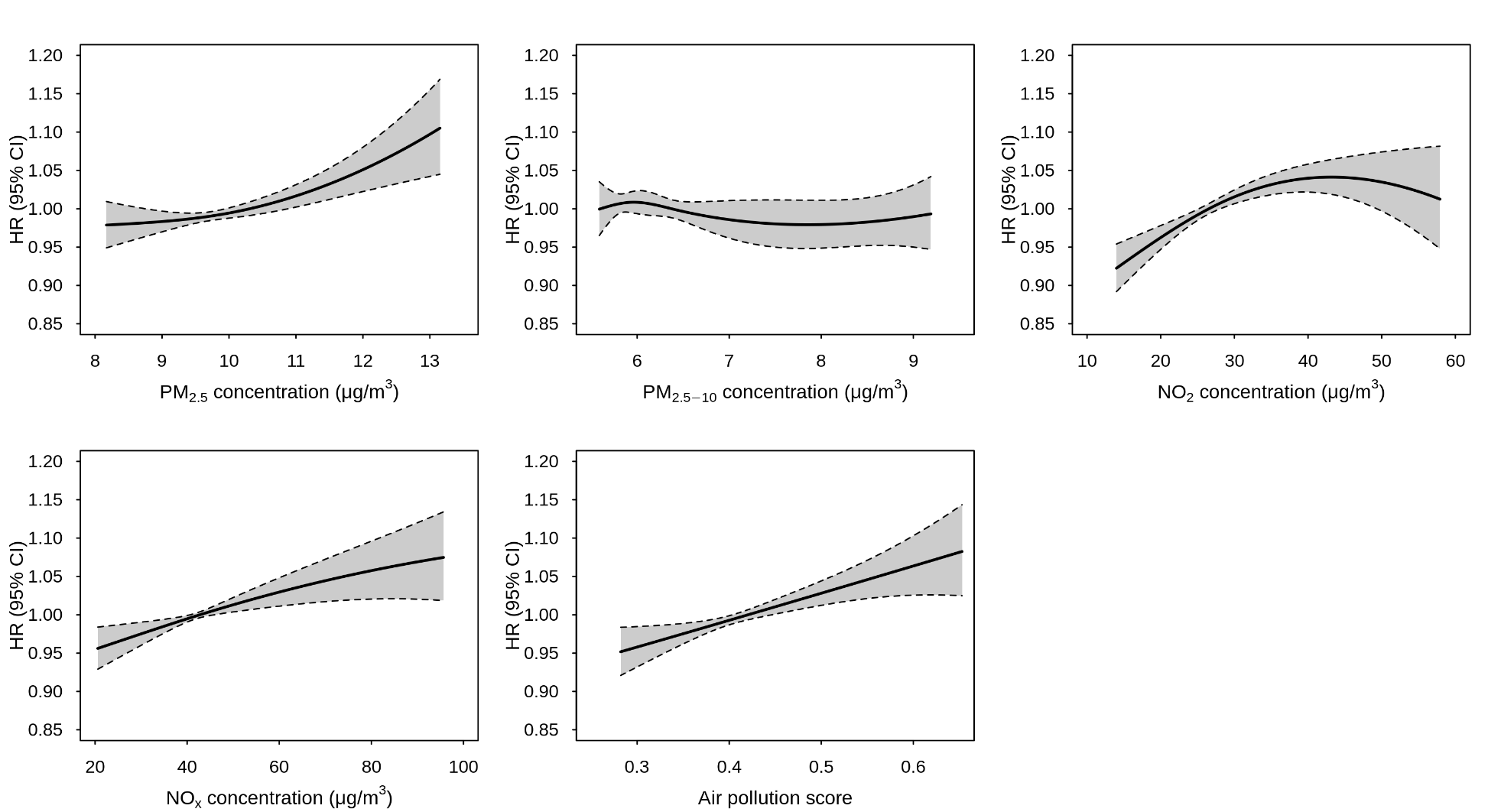
Fig. S2.** Exposure-response curves for the relationships between air pollution and incidence of atrial fibrillation. The gray areas denote 95% confidence intervals. Abbreviations same as in Table S3.

**
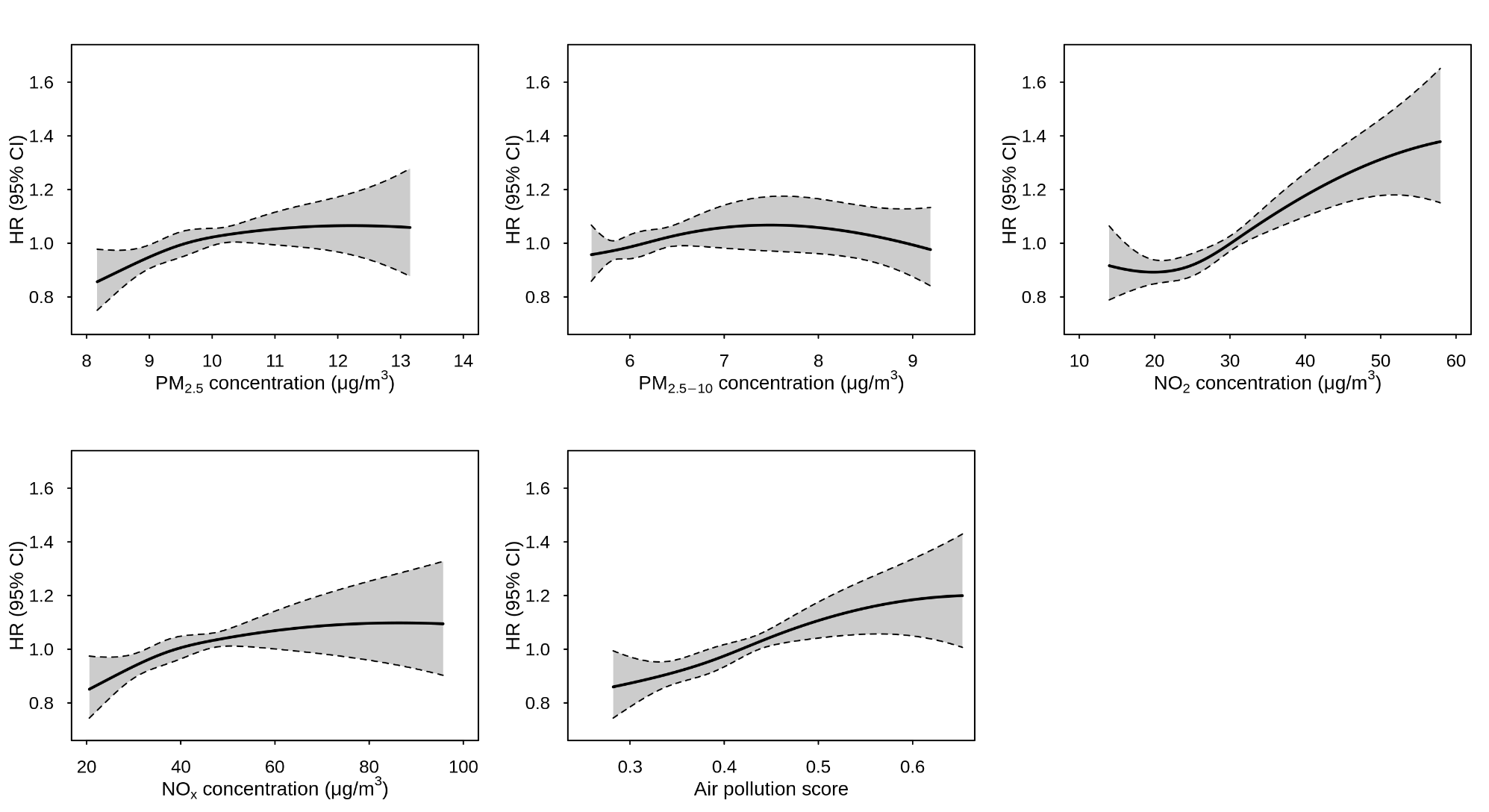
Fig. S3.** Exposure-response curves between air pollution and incidence of supraventricular tachycardia. The gray areas denote 95% confidence intervals. Abbreviations same as in Table S3.

**
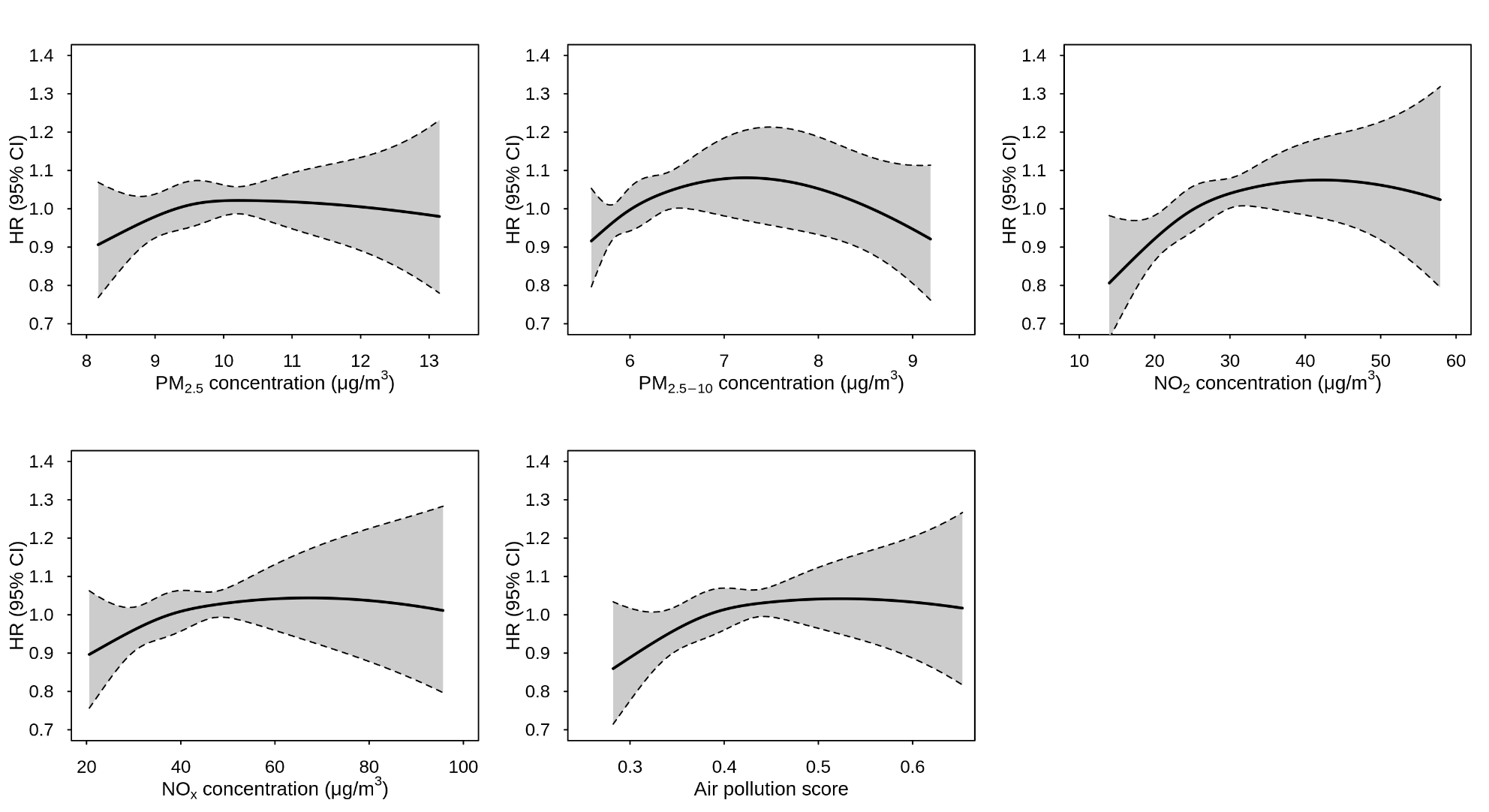
Fig. S4.** Exposure-response curves between air pollution and incidence of ventricular fibrillation and ventricular tachycardia. The gray areas denote 95% confidence intervals. Abbreviations same as in Table S3.

**
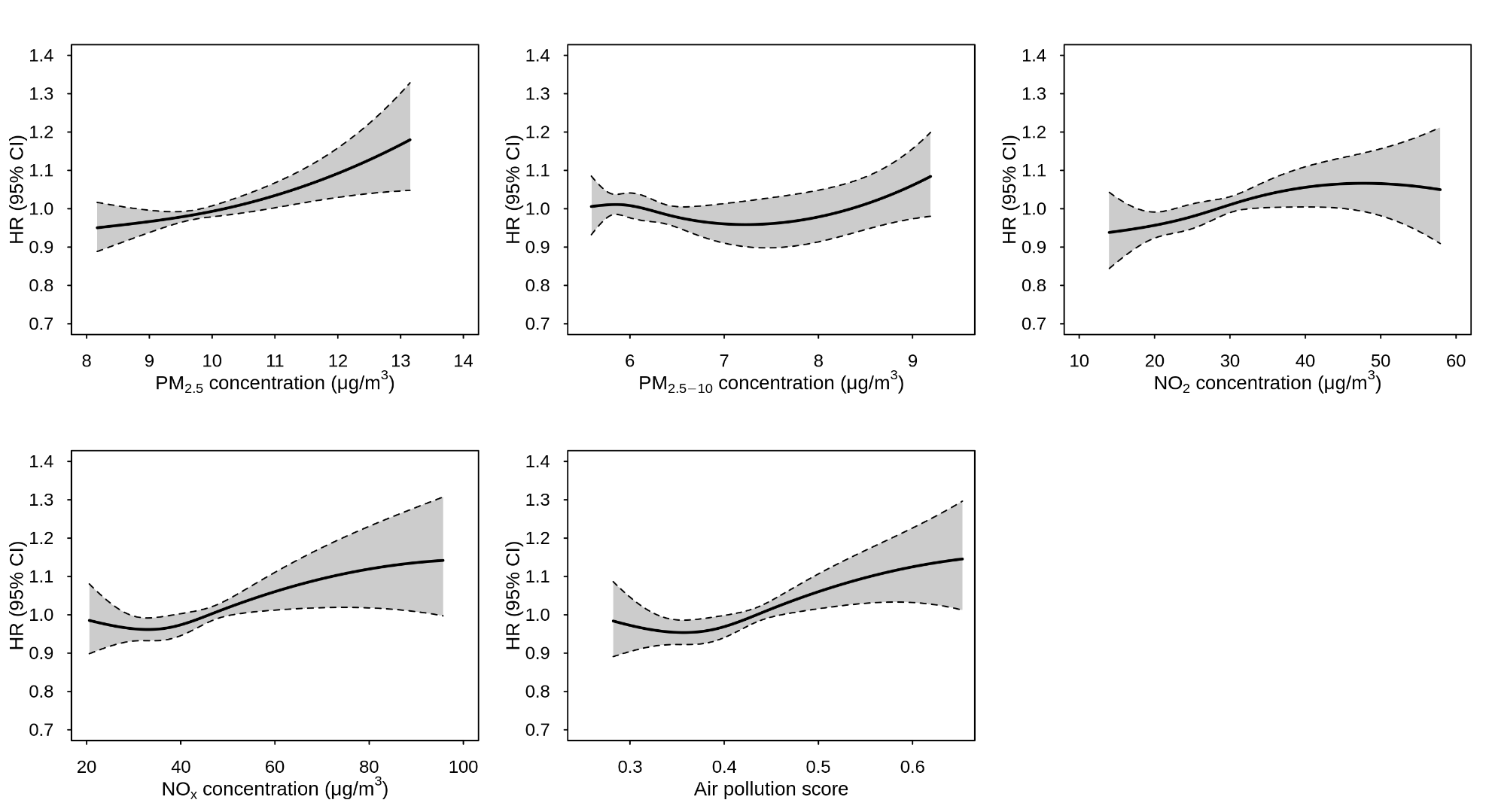
Fig. S5.** Exposure-response curves between air pollution and incidence of atrioventricular block. The gray areas denote 95% confidence intervals. Abbreviations same as in Table S3.

**
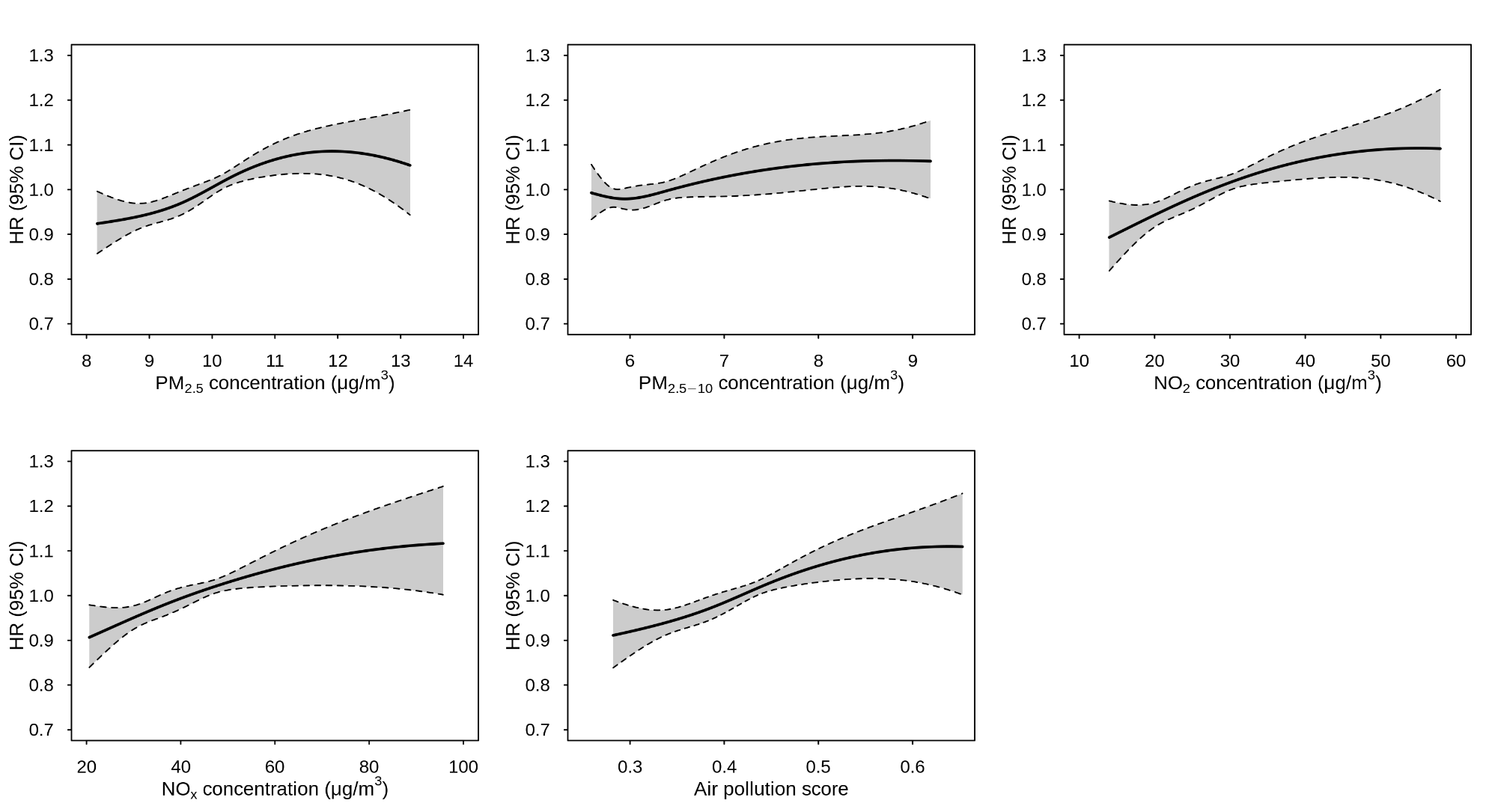
Fig. S6.** Exposure-response curves between air pollution and incidence of intraventricular block. The gray areas denote 95% confidence intervals. Abbreviations same as in Table S3.

**
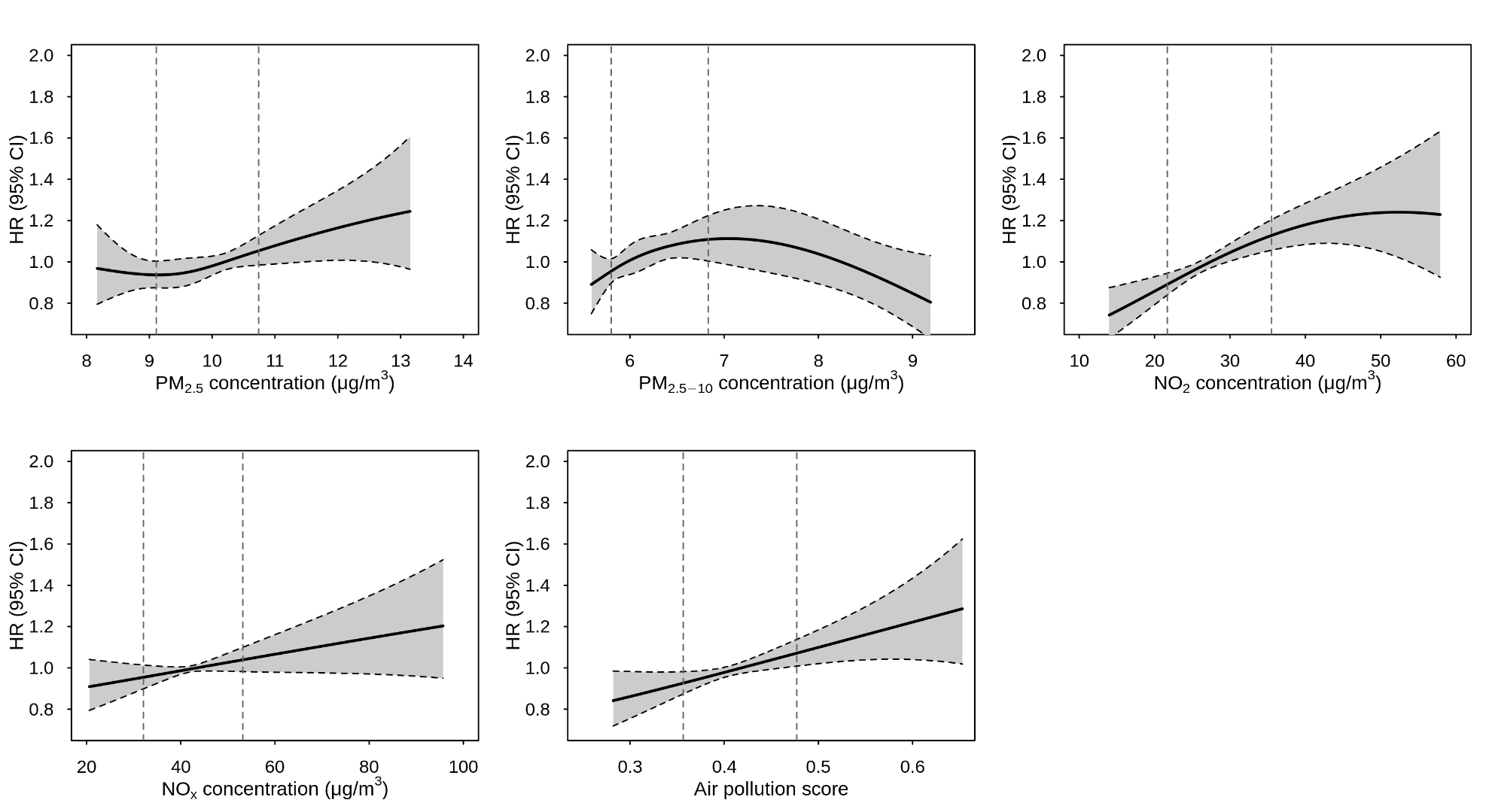
Fig. S7.** Exposure-response curves between air pollution and incidence of ventricular premature beats. The gray areas denote 95% confidence intervals. Abbreviations same as in Table S3.
